# Is hypothermia more neuroprotective than avoiding fever after cardiac arrest?

**DOI:** 10.1093/cvr/cvab297

**Published:** 2021-10-27

**Authors:** Erik Sveberg Dietrichs, Rachel Myles, Godfrey Smith

**Affiliations:** 1 Experimental and Clinical Pharmacology Research Group, Department of Medical Biology, UiT, The Arctic University of Norway, Hansine Hansens vei 14, N-9037 Tromsø, Norway; 2 Center for Psychopharmacology, Diakonhjemmet Hospital, Forskningsveien 13, N-0373 Oslo, Norway; 3 Institute of Cardiovascular & Medical Sciences, University of Glasgow, West Medical Building, Glasgow G12 8QQ, UK

**Keywords:** Hypothermia, Cardiac arrest, Targeted temperature management, Therapeutic hypothermia, QRS/QTc

Hypothermia was increasingly proposed as a neuroprotective therapy in the 1990s, culminating in the publication of two randomized trials in 2002, which showed beneficial effects of therapeutic hypothermia in comatose survivors of cardiac arrest.^1^^,^^2^ Therapeutic hypothermia was subsequently included in guidelines for the treatment of this patient group. However, doubt as to whether hypothermia provided benefit over targeted normothermia (i.e. preventing fever) was raised in 2013 when a large randomized trial showed no difference in survival or neurological function between cardiac arrest survivors cooled to 33 or 36°C.^3^ Recently the results from a large (1900 patients) randomized open-label trial, comparing patients cooled to 33°C or maintained normothermic (≤37.5°C), was published by Dankiewicz *et al.*^4^ in the *New England Journal of Medicine*. In agreement with the 2013 publication,^3^ no difference in disability or death was detected between the treatment groups after 6 months. The only significant difference in adverse events was arrhythmias; those resulting in haemodynamic compromise were more common in the hypothermia group than in the normothermia group (24% vs. 17%).^4^

Preclinical studies strongly suggest that lowering core body temperature is neuroprotective. In a systematic review of hypothermia and neuroprotection, 47 of the 55 included experimental animal studies reported a neuroprotective effect of hypothermia.^5^ From patients undergoing aortic arch surgery, studies show that cooling to below 20°C allows prolonged cardiac standstill without neurological sequela, compared with patients undergoing swift surgery at normothermia with shorter periods of cardiac standstill.^5^ A reduction in cerebral metabolism is a key factor. For every 1°C of temperature reduction, brain glucose turnover decreases 5%.^5^ Cooling can further affect a number of the mechanisms causing ischaemic brain injury. Accumulation and release of excitotoxic amino acids are reduced, as well as harmful calcium influx into cells. Inhibition of inflammatory responses are also involved in the protective effect.^5^

Based on our understanding of hypothermia neuroprotection from preclinical studies, there is a potential advantage to the patient if hypothermia were induced before or coincident with an ischaemic event. The standardized protocol of experimental animal models allows detection of small differences between hypothermic and normothermic groups and cooling is often initiated shortly after the incident.^5^ But cooling within minutes is seldom possible in cardiac arrest patients. Interestingly, some experimental evidence does suggest that neuroprotection could be provided hours after the incident. Selective cooling of the brain to 25°C, 2.5 h after cerebral arterial occlusion in baboons, reduced infarct size to 0.5%, compared with 35% in normothermic animals.^6^ This is still more rapid cooling than is seen in clinical practice, where even in the setting of a clinical trial the window for inclusion was set at 3–4 h after return of spontaneous circulation (ROSC),^3^^,^^4^ which by definition will be some time after the onset of cerebral ischaemia.

Accordingly, it appears optimal to cool as early as possible to gain maximal benefit from the neuroprotective mechanisms shown in experimental animal studies. An important question is therefore whether it is practicable to cool comatose survivors of cardiac arrest soon enough to provide neuroprotection. The results presented by Dankiewicz *et al.*^4^ suggest that either this is not currently achievable in routine clinical practice and/or the potentially detrimental effects of hypothermia might outweigh any benefits of neuroprotection.

In several recent publications, we have explored the electrophysiological effects of hypothermia on the heart.^7–10^ The data demonstrate that temperatures used for therapeutic hypothermia after cardiac arrest are pro-arrhythmic compared to normothermia, or interestingly, to more severe hypothermia. When the temperature approaches 30°C, prolongation of ventricular repolarization occurs in the presence of normal conduction, causing increased repolarization heterogeneity and lowering of ventricular fibrillation (VF) threshold.^7–10^ Whether this intrinsic effect on cardiac electrophysiology was responsible for the excess of arrhythmias associated with haemodynamic compromise reported by Dankiewicz *et al.* merits consideration. In their study, cardiovascular instability and arrhythmias were also the most common reason for rewarming before the therapeutic hypothermia protocol was completed.^4^

It is apparent that effective therapeutic hypothermia treatment after cardiac arrest, and it is questionable whether patients should be selected for therapeutic hypothermia several hours after ROSC. It remains to be established whether earlier or more rapid cooling could be beneficial. Identification of patients at risk of arrhythmias would potentially increase the safety of targeted temperature management and use of therapeutic hypothermia. For instance, an electrocardiogram-marker like QRS/QTc^9^^,^^10^ could be used as a tool to select patients suitable for cooling or to monitor the cardiac effects of cooling to predict risk of ventricular arrhythmias.

This is a patient group with extremely poor clinical outcomes. Dankiewicz *et al.* reported 50% mortality at 6 months and high rates of at least moderately severe disability in survivors. These figures are stark and underline the importance of continued efforts to harness the neuroprotective effects of hypothermia in routine clinical practice. Alongside clinical trials, research into the links between hypothermia and arrhythmia and the optimal timing and implementation of hypothermia may still allow this goal to be realized.

**Conflict of interest:** The authors declare that there is no conflict of interest.

## Figures and Tables

**Figure:**
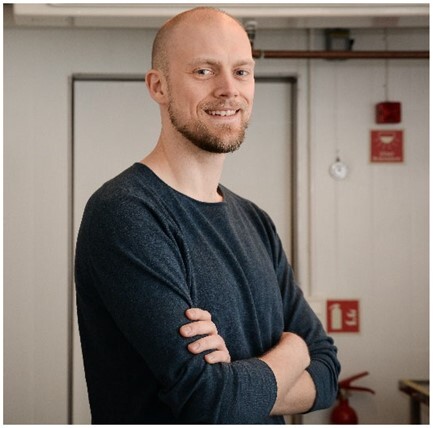


**Figure:**
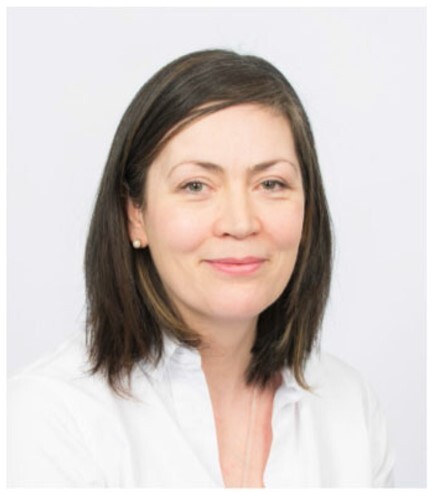


**Figure:**
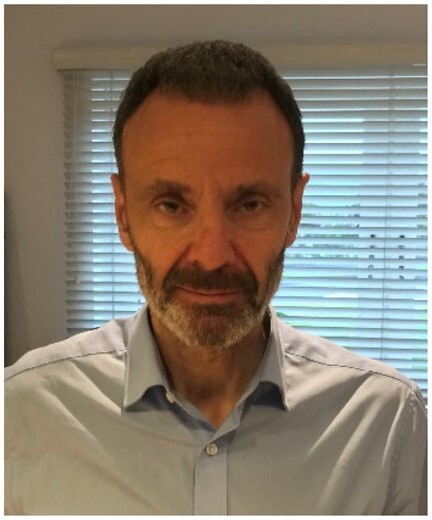


## Authors

**Biography:** Dr Erik Sveberg Dietrichs is Consultant Physician at the Centre for Psychopharmacology, Diakonhjemmet Hospital in Oslo and Associate Professor in Clinical Pharmacology at UiT, The Arctic University of Norway. He is head of the Experimental and Clinical Pharmacology research group at the same university. Dietrichs is currently serving his second term as a national board member in The Norwegian Association of Physicians in Science. He is also an author of three published popular science books and editor of the Psychopharmaca section of the Norwegian Encyclopaedia. His research addresses psychiatric and cardiovascular pharmacology in general, with special focus on the effect of drugs and hypothermia on cardiovascular physiology, both in experimental models and clinical studies.

## Notes

**Biography:** Dr Rachel Myles is a clinical academic electrophysiologist based in Glasgow, UK. Rachel trained in Oxford, London, Edinburgh, Glasgow, and California before becoming a consultant and starting her own research lab at the University of Glasgow, supported by a Wellcome Trust Fellowship. Rachel divides her time between her clinical practice in heart rhythm disorders and translational research into the mechanisms of arrhythmias. Her group’s research focuses on the expression of cellular behaviour in the whole heart and how this generates ventricular arrhythmia in ischaemic heart disease and inherited channelopathies.

## Notes

**Biography:** Godfrey Smith is a Professor of Cardiovascular Physiology and currently deputy director of the Institute of Cardiovascular and Medical Sciences at Glasgow University. He has served on several national and international committees, including the Physiological Society and the European Society of Cardiology. Godfrey holds honorary appointments at Imperial College London and Trondheim University Norway. His research addresses the cardiac excitation contraction process in health and disease including mechanisms of contraction failure; small molecule modulators of Ca signalling; and arrhythmic mechanisms associated with ischaemic cardiomyopathy. His research uses a series of novel biophysical techniques including multiphoton microscopy. Smith is a cofounder and acting CSO of a spin-out company (Clyde Biosciences Ltd) centred on medium through-put functional cardiotox assays using on novel optical techniques.
